# *Bacillus cereus* Toxins

**DOI:** 10.3390/toxins13050295

**Published:** 2021-04-21

**Authors:** Erwin Märtlbauer, Per Einar Granum

**Affiliations:** 1 Department of Veterinary Sciences, Faculty of Veterinary Medicine, Ludwig-Maximilians-University Munich, 85764 Oberschleißheim, Germany; 2 Department of Paraclinical Sciences, School of Veterinary Science, Norwegian University of Life Sciences, 1430 Aas, Norway; per.einar@gmico.no

*Bacillus cereus sensu stricto* is an important pathogen causing food poisoning, as well as extraintestinal diseases. It presents a major challenge for the food industry, as it can easily be spread to different foods via crop plants and livestock. Furthermore, its ability to produce biofilms and spores makes *B. cereus* extremely resistant towards cleaning and disinfection procedures, as well as the technological processing of foods. The bacterium is estimated to be responsible for 1.4–12% of all food poisoning outbreaks worldwide [1]. In the European Union, bacterial toxins (*Clostridium*, *Staphylococcus,* and *B. cereus*) accounted for 17.7% (2016) and 15.9% (2017) of all registered food- and water-borne outbreaks [2,3]. The emetic form of disease, namley food intoxication, is caused by the heat-stable cyclic dodecadepsipeptide cereulide, manifesting as nausea and vomiting as soon as half an hour after consumption of the contaminated food, and, although rare, even fatal outcomes due to liver failure [4,5]. The diarrheal form emerges from food infections with enteropathogenic strains, also known as toxico-infections, in which the toxins are produced by viable bacteria in the human intestine. The main symptoms are diarrhea and abdominal cramps, which manifest after approximately 8–16 h. The three main, pore-forming protein enterotoxins are the tripartite hemolysin BL (Hbl) and non-hemolytic enterotoxin (Nhe), as well as the single protein cytotoxin K (CytK) [4,6,7,8].

This Special Issue focuses on the knowledge gained especially on cereulide and the enterotoxins, but also on other virulence factors. The overarching goal is the holistic and reliable risk assessment of contaminated foods—where detailed knowledge on the prevalence of the bacteria and their toxin genes in foods, the course of the diarrheal disease, the structures and mechanisms of action of the toxins, and the individual role of further virulence factors is essential.

Because of its ubiquitous nature and the formation of highly adhesive and resistant endospores, *B. cereus* appears in a great variety of different foods. While emetic strains are mainly associated with starchy foodstuffs such as rice and pasta, enteropathogenic isolates are detected in all kinds of foods, including milk products, vegetables, meat products, sauces, soups, puddings, spices, poultry, and sprouts. The numerous studies performed on its prevalence and survival in different foods, as well as on the distribution of its toxin genes, are summarized in two reviews belonging to this Special Issue [9,10]. Additionally, Sánchez-Chica et al. determined the presence and toxigenic and genomic diversity of *B. cereus sensu lato* isolates of a yet barely investigated raw material, cassava starch, from bakeries and powdered food companies in Colombia. *B. cereus s. l.* was found in 57% of all samples. Besides a broad genomic diversity, 12 toxigenic profiles were detected, with the *nheABC* operon being the most prevalent and the *cesB* gene not found [11]. This study underlines the great genomic and enterotoxigenic diversity, as well as the importance of determining *B. cereus s. l.* presence in different foods. Moreover, a third review focuses on the increasing prevalence rate and the distribution of *B. cereus* in dairy products in China. Not only the epidemiological characteristics are summarized, but also detection techniques for the pathogen and its toxins, providing insight into the implementation of intervention strategies and therapeutic options [12]. This thematic field is complemented by another overview article about the risk of contamination of human breast milk (HBM). *B. cereus* is the most frequent cause for discarding HBM, as it can induce severe infections in newborns, which are rare, but sometimes fatal. *B. cereus* infections in neonates and the toxins involved are reported, as well as the detection and decontamination procedures for risk reduction [13].

One of the reviews pursues the complex infection procedure with enteropathogenic *B. cereus* in detail. As only enterotoxins secreted from viable bacteria inside the host intestine contribute to the disease, this toxico-infection must be seen as a multifactorial process. This includes—next to prevalence and survival in different foodstuffs—the survival of the stomach passage, germination of spores, enterotoxin production under intestinal conditions, and the influence of consumed foods and the intestinal microbiota [9]. Detailed knowledge of these single steps is the basis for reliable virulence analysis and risk-oriented diagnostics for enteropathogenic *B. cereus*.

Nevertheless, the course of the diarrheal disease is, to a great extent, determined by the production and action of the enterotoxins in the intestine. In a comprehensive review, 30 years of work on the food as well as cereulide and CytK. The latest finding on the genetic organization, the highly variable gene expression and toxin secretion, the mode of action, and the effects on target cells are shown [10]. Of special interest is the mode of pore formation of the tripartite enterotoxins Hbl and Nhe. Hbl comprises the proteins L1 and L2 and the binding component B, while Nhe consists of the proteins NheA, NheB, and NheC. Both enterotoxins need a specific binding order and a defined concentration ratio of their protein components for optimal pore formation. So far, the crystal structures of HblB and NheA have been resolved, and these data indicate that Hbl and Nhe are members of the alpha helical Cytolysin A family of pore forming toxins. Additionally, Worthy et al. solved the crystal structure of Hbl L1, which shows an extended hydrophobic beta tongue region that may be involved in pore formation. Using molecular docking, possible interactions between Hbl L1 and Hbl B were predicted [14]. Complex formations between NheB and C, Hbl B and L1, and Hbl L1 and L2 in solution have been observed before. Another research article describes the characteristics of the Hbl protein complexes. Distinct complexes of up to 600 kDa were found. Their structure and size were primarily defined by Hbl B. Heat treatment proved to release Hbl B from the tight complexation, resulting in enhanced pore forming and cytotoxic activity. Furthermore, Hbl-induced pores were shown for the first time, and appeared to be rather small and instable, as well as cation-selective [15].

Besides the enterotoxins and their fascinating mode of action, this Special Issue also focuses on methods for toxin detection and for the prevention of illness, as well as on the contributions of further putative virulence factors to the disease. On the one hand, these topics are summarized in the participating reviews [9,10,12,13]. On the other hand, one research work introduces reversed phase chromatography as a suitable method for the detection and relative quantification of cereulide with common equipment in microbiological and biochemical research laboratories [16]. In the research article by Tran et al., the cell wall peptidase CwpFM from pathogenic *B. cereus* strains shows a specific stretch of 20 disordered residues, suggesting this protein as a potential marker for differentiation between pathogenic and apathogenic *B. cereus* strains, as well as for improved food safety [17].
